# Enhancement of the Anti-Skin Wrinkling Effects of *Aloe arborescens* Miller Extracts Associated with Lactic Acid Fermentation

**DOI:** 10.1155/2020/2743594

**Published:** 2020-06-02

**Authors:** Hyang Seon Ro, Hyun Jun Jang, Gyu Rae Kim, Sang Jin Park, Hyeon Yong Lee

**Affiliations:** ^1^Department of Chemical and Biochemical Engineering, College of Engineering, Dongguk University, 30 Pildong-ro 1 gil, Jung-gu, Seoul 04620, Republic of Korea; ^2^R&D Center, NOWCOS Ltd., A-1004 BYC Highcity, Gasan Digital 1, Seoul 08506, Republic of Korea; ^3^R&DB Center, Beauty Science Ltd., 220 Gasangil, Sejong 30003, Republic of Korea; ^4^Department of Medical Biomaterials Engineering, College of Biomedical Science, Kangwon National University, Kangwon University Road 1, Chuncheon 24341, Republic of Korea

## Abstract

This work clearly shows that *Aloe arborescens* but not gels from *Aloe vera*, a common juice-type product of Aloe, exerted anti-skin wrinkling effects, and these effects were greatly enhanced by lactic acid fermentation with *Lactobacillus plantarum*. Treatment with the extract from the fermentation process (FE) at a dose of 0.5% highly activated human fibroblast cells by up to 175%, whereas 140% activation and 105% activation were observed with the extract obtained using conventional water extraction (WE) and the gel from *A. vera* (GE), respectively. The treatment of human fibroblasts with FE at a dose of 0.5% increased collagen production by up to 170% and inhibited MMP-1 synthesis to 48%, which is likely due to its high antioxidant activity because the WE and GE showed markedly lower effects compared with those of the FE. Interestingly, the FE exhibited a profile dominated by relatively low-molecular-weight (MW) polysaccharides: 20% of the total polysaccharides in the FE were in the MW weight range of 600 to 900, whereas 95% of the total polysaccharides in the GE were in the MW range of 200,000 to 300,000. This result suggests that the larger polysaccharide molecules in the extract might be broken down during lactic acid fermentation, and the easy penetration of the small molecules in the extract into fibroblast cells thus results in improved anti-skin wrinkling effects. This conclusion is also supported by the finding that the FE and WE, but not the GE, contained similar amounts of barbaloin, a strong antioxidant eluted from *A. arborescens* through the fermentation process. Therefore, this study strongly indicates that the enhanced anti-skin wrinkling effects of the FE are most likely due to synergistic effects between the barbaloin and the low-MW polysaccharides retained after the fermentation process.

## 1. Introduction

More than approximately 650 species of aloe exist on Earth and have been widely used for a long time due to their various effects, such as anti-gastric ulcer, anticonstipation, antiburn, and anticancer activities, immune activation, and ability to treat skin disorders [1–4]. Various types of functional foods from aloe, such as laxatives, immune foods, and supplementary foods for various patients that contain many bioactive substances, including many types of polysaccharides (mainly glucomannan), flavonoids, anthraquinone, gibberellin, *β*-sitosterol, and pyrones, have also been introduced in recent years [5–7]. Interestingly, based on its activities, aloe is considered particularly effective for the treatment of various diseases related to skin disorders, such as atopic dermatitis, and skin inflammation and for wound healing and enhancing skin regeneration [8–11], and these treatment effects are likely due to the complex effects of various polysaccharides and anthraquinones, such as barbaloin and aloin [12–14]. However, to date, only six to seven species of the aloe family, including *Aloe vera* Linne, *Aloe barbadensis* Miller, *Aloe arborescens* Miller, *Aloe saponaria* Haw, and *Aloe perryi* Baker, have been consumed by human beings as functional foods or pharmaceuticals, and these uses depend on the origin of the species [15]. Among the related substances, gels obtained from peeling off the skin of *A. vera* Linne have been the most studied. However, these gels contain less than 5% solids, and the rest of the gels are composed of water; thus, fairly large quantities of aloe gels are required to achieve efficacy [16]. Moreover, most of the solids consist of very large polysaccharide molecules, which results in inherent difficulties in their processing for industrial applications and the requirement of more processes for peeling off the skins [17]. Compared with the limitations in the processing of gels from *Aloe vera*, *A. arborescens* has handling-related advantages due to their relatively small body and small amounts of juice in the leaves, which allows the processing of the whole bodies of *A. arborescens* [18]. However, even though its biological activities are similar to those of *A. vera* Linne, *A. arborescens* has not been actively employed due to its bitter taste and low production yields [19–21].

Therefore, the development of a technique for the processing of *A. arborescens* that does not affect its activities and maintains the stability of large-molecular-size polysaccharides is necessary. For example, a lactic acid fermentation process could be employed for the processing of *A. arborescens* under low temperatures and mild extraction conditions, which would not deteriorate the bioactive substances and maintain the stability of the polysaccharides in the extracts [22, 23]. Moreover, the lactic acid fermentation process improves the elution yields of active components from natural resources and catalyzes the bioconversion of the metabolites, which can increase the efficacies based on the synergistic effects of various components in the extracts [23, 24]. For these purposes, *Lactobacillus* families have mostly been used because they are considered generally recognized as safe (GRAS) by the FDA for food purposes [25] and cosmetic applications [26–28], which means that the fermentation of *Lactobacillus* species could be safe and easily adopted for various foods, cosmetics, and drugs. As a result, previous studies have intensively investigated the fermentation of *Lactobacillus* species. For many cosmetic applications, the anti-skin wrinkling effects of natural products have been of interest because skin wrinkling is a complex process that involves the collapse of the extracellular matrix of the skin along with skin barrier dysfunction and skin aging, which are one of the most urgent problems to be resolved [28, 29].

In this study, the fermentation process of *A. arborescens* with lactic acid bacteria is introduced to improve the cosmeceutical activities of this species. More specifically, the anti-skin wrinkling effects and the reduced toxicity of *A. arborescens* fermented with *Lactobacillus plantarum* were investigated because this lactic acid bacterium exhibits a relatively high ability to enhance the biological activities of *A. arborescens* in many applications compared with other *Lactobacillus* species [30, 31]. Therefore, this work could provide useful information for expanding the use of aloe in cosmetic applications.

## 2. Materials and Methods

### 2.1. Preparation of the Extracts

As the control, leaves of fresh *Aloe vera* Linne (grown and harvested at Kimjeonmoon Aloe Farm in Jeju, Korea, 2019) were peeled off, extracted with a pressure extractor (U350, Ilsung Co., Incheon, Korea) to obtain gels from the inner parts of the aloe plants, and lyophilized to obtain a powder (GE). For the preparation of the *A. arborescens* Miller extract, 100 g of dried whole aerial parts of *A. arborescens* Miller (grown and harvested at Kimjeonmoon Aloe Farm in Jeju, Korea, 2019) was extracted with distilled water (1 : 10, w/v) at 100°C for 24 hours using a vertical reflux condenser (TL-6, Misung Scientific, Yangjoo, Korea). The extracts were then centrifuged at 9,000g and room temperature for 30 min, and the supernatants were condensed using a rotary vacuum evaporator (EV-101, Eyela, Tokyo, Japan). The concentrates were subsequently freeze-dried with a lyophilizer (PVTFA, 10AT, IlShin, Suwon, Korea) to obtain the powder for the experiments (WE).

For lactic acid fermentation, 5% (v/v) of 1 × 10^6^ cells/mL *Lactobacillus plantarum* (14917, ATCC, Manassas, VA, USA) were inoculated into MRS culture medium (Sigma, St. Louis, MO, USA) with 10% (w/v) ground *A. arborescens* Miller powder in a 1-L top-closed jar and anaerobically grown for 4 days at 37°C in a shaking incubator (KB-105, Korea Biotech., Seoul, Korea) with a shaking speed of 100 rpm. The pH of the culture medium was measured every day to monitor the progress of the fermentation, and the cultivation was terminated when the pH of the culture medium reached a value of less than 3.5. The culture was then further extracted with an ultrasonicator (AUG-R3-900, Asia Ultrasonic, Gyeonggi-Do, Korea) at a fixed frequency of 40 kHz with an input power of 1500 W for 1 hour at room temperature, and the culture broth was centrifuged at 10,000g and room temperature for 30 min. The supernatant was concentrated with a rotary vacuum evaporator and freeze-dried (FE). All of the extracts were stored at −20°C before use.

### 2.2. Measurement of the Barbaloin Contents in the Extracts

For the measurement of the barbaloin contents in the samples, 5 g of the extract powder was mixed with 5 mL of methanol, and after 75 mL of distilled water was added, the mixture was boiled for 30 min. The solution was then filtered using filter paper, and 200 mL of water was added to the supernatant. Subsequently, 10 mL of this solution was mixed with 1 mL of FeCl_3_ and 6 mL of HCl and distilled vertically with a vertical reflux condenser for 4 hours. The condensed solution was extracted three times with 4 mL of 1 N NaOH and 20 mL of CCl_4_ in a funnel. The fraction of CCl_4_ was collected and washed with 10 mL of water, and 100 mL of CCl_4_ solution was added. Subsequently, 20 mL of this solution was evaporated in a water bath at 80°C, and the residue was mixed with 10 mL of 0.5% methanol magnesium acetate solution. The absorbance of the solution was measured using a microplate reader (EMax ELISA Reader, Sunnyvale, CA, USA) at a wavelength of 512 nm using methanol as the standard. The quantity of barbaloin was calculated using the following:

(1) $$\begin{array}{c} \text{concentration of anhydrous barbaloin }\left(\%\right)=\left(\frac{A}{240}\right)\times 10\times 200\times \left(\frac{1}{S}\right), \end{array}$$

where *A* is the absorbance at 512 nm and *S* is the weight of the sample (g) [32, 33].

### 2.3. Determination of the Profiles of Polysaccharides of Different Molecular Weights (MWs) in the Extracts

To assess the MW distributions of the polysaccharides in the three samples, matrix-assisted laser desorption ionization-time of flight (MALDI-TOF) MS analyses were performed as follows [34]: 1 *μ*L of the mixture and 1 *μ*g of the sample were dissolved in 20 *μ*L of DMSO and loaded onto a MALDI-TOF instrument (Autoflex III speed TOF/TOF, Bruker Daltonics, Bremen, Germany) with a smart beam laser as the ionization source under linear-gradient conditions over an m/z range of 350 to 300,000. All spectra were acquired in the positive mode as the matrix at 19 kV with an accelerating voltage of 50 Hz and repetition rate, and the peaks were analyzed using an alpha-cyano-4-hydroxycinnamic acid (CHCA) matrix according to the standard protocols provided by the manufacturer [34, 35].

### 2.4. Assessment of the Cellular Cytotoxicity of the Extracts

The cytotoxicity of the extracts was determined using the 3-(4,5-dimethythiazo-2-yl)-2,5-diphenyltetrazolium bromide (MTT) assay as follows [36]: 1.0 × 10^4^ human fibroblast cells/well (CCD-986sk, ATCC, Manassas, VA, USA) were inoculated into a 96-well plate and further incubated in a CO_2_ incubator at 37°C for 6 hours. Subsequently, 200 *μ*L of the three extracts (GE, WE, and FE) with various concentrations was added to the cells, and the cells were cultured for 24 hours. MTT solution (5 *μ*g/mL) was added to each well, and the supernatant was removed 4 hours later. Thereafter, 10 *μ*L of acid-isopropanol (0.04 N HCl in isopropanol) was added to the solution, and the absorbance of the solution was measured using a microplate reader at a wavelength of 565 nm. The cell cytotoxicity was expressed as the percent ratio of the cell number in the sample treated with a certain concentration to the cell number in the untreated control sample [37].

The morphology and density of the fibroblast cells treated with the various extracts at doses of 0.01% and 0.5% were also observed under an inverted microscope (IMT-2, Olympus, Tokyo, Japan) and compared with those of the control (untreated cells).

### 2.5. Measurement of the Antioxidant Activities of the Extracts

The antioxidant activities of the extracts were measured based on the 2,2 diphenyl-1-picrylhydrazyl (DPPH) free radical scavenging activities according to the Dietz method [38]: 80 *μ*L of the extract (GE, WE, and FE) or 80 *μ*L of 0.05% (w/v) Trolox (Sigma, St. Louis, MO, USA) as a positive control was mixed with 200 *μ*L of 0.1 mM DPPH dissolved in ethanol in a 96-well plate and left in a dark room at 25°C for 20 min. The absorbance of the mixture at 525 nm was then measured using a microplate reader. The DPPH free radical scavenging activity (%) of each sample was estimated as the ratio of the difference in absorbance between the control and the sample to the absorbance of the control and is expressed as the IC_50_ to reflect the concentration that results in 50% of the activity of the control:

### 2.6. Estimation of the Production of Collagen from Human Fibroblast Cells by the Extracts

The production of collagen by human fibroblast cells (CCD-986sk) was measured using a Procollagen Type 95 I C-peptide (PIP) EIA Kit (Takara, Otsu, Japan) based on the following protocol [39]: 100 *μ*L of the antibody-POD conjugate solution was added to the plate provided with the kit, and 1.0 × 10^5^ viable cells/mL were cultivated with 20 *μ*L of the sample at different concentrations for 24 hours. 0.1 ng/mL transforming growth factor-*β*1 (TGF-*β*1, Sigma, St. Louis, MO, USA) was used as a positive control. The cells were further cultured for 3 hours at 37°C in the dark. The culture medium was removed, and the cells were washed three times with 400 *μ*L of washing buffer. The substrate solutions provided with the kit were then added to each well, and the plate was allowed to stand for 15 min at room temperature. The reaction was subsequently stopped by the addition of 100 *μ*L of 1 N H_2_SO_4_ (stop solution) to each well and shaking for 1 minute. The optical density of each well at 450 nm was then measured using a microplate reader and quantified through a linear regression curve obtained for the standard provided with the kit in accordance with the manufacturer's recommended protocols.

### 2.7. Measurement of the Inhibition of Matrix Metalloproteinase-1 (MMP-1) Synthesis by the Extracts

First, 1.0 × 10^5^ human fibroblast cells/mL (CCD-986sk) were inoculated into a 96-well plate and allowed to grow for 24 hours in an incubator with 5% CO_2_ at 37°C. After the medium in each well was removed, various concentrations of the extracts and 0.1% (w/v) retinol (transretinol, Sigma, St, Louis, MO, USA) as a positive control in the fresh medium were added to the wells, and the cells were further incubated for 48 hours. Subsequently, 100 *μ*L of the medium was transferred into a Human MMP-1 ELISA Kit (RayBiotech, Norcross, GA, USA) and reacted for 2 hours at room temperature. After the reaction, the medium was removed from the wells, the wells were washed four times with PBS buffer, and 100 *μ*L of MMP-1 detection antibody was added to the wells. The plates were further incubated for 1 hour at room temperature, and the medium in the wells was removed. The plates were washed again with PBS buffer, 10 mL of streptavidin solution was added, and the plate was then shaken for 45 min at room temperature. The medium in the wells was removed, and the plate was washed twice with PBS buffer. Subsequently, 100 *μ*L of substrate reagent was added to the wells, and the plate was incubated at room temperature without light for 30 min. The reaction was stopped by the addition of 50 *μ*L of stop solution. The inhibition of MMP-1 production was measured by comparing the absorbance of the samples in each well at 450 nm with a linear regression curve obtained using the standard provided with the kit [40]. All of the procedures were in accordance with the protocols recommended by the manufacturer.

### 2.8. Statistical Analysis

All of the experiments were repeated three times, and the results are expressed as the means ± standard deviations (SDs). The statistical significance of the difference was assessed by one-way analysis of variance (ANOVA) (SAS 9.1, SAS, Cary, NC, USA).

## 3. Results and Discussion

### 3.1. Cellular Cytotoxicity and Antioxidant Activities of the Extracts

**Figure 1:**
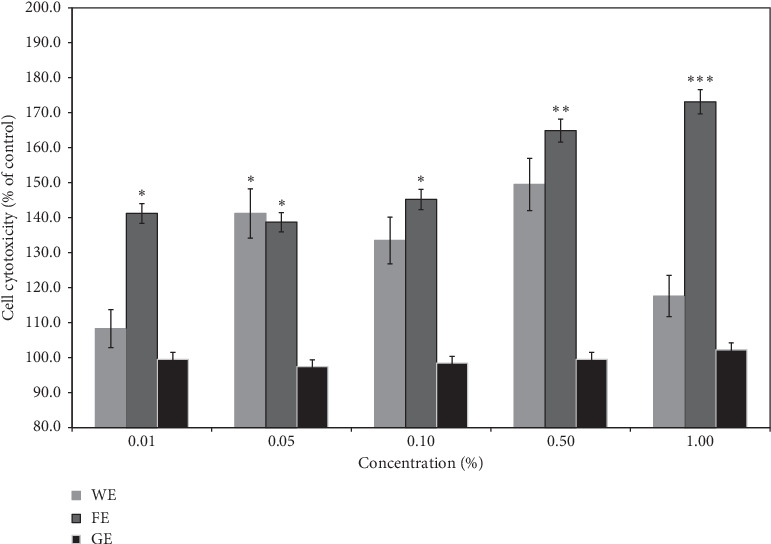
Cytotoxicity of the aloe extracts from the three different processes against human fibroblast cells. GE, gel juice extracted after peeling off the leaves of *A. vera*; WE, the water extract from the whole aerial parts of *A. arborescens* at 100°C for 24 hours; FE, fermentation of the whole aerial parts of *A. arborescens* grown with *L. plantarum* at 37°C for 4 days and further extracted by an ultrasonicator at 40 kHz for 1 hour at room temperature. Error bars are mean values ± SD from triplicate separate experiments. The significance of difference was set to ^*∗*^*p* < 0.05, ^*∗∗*^*p* < 0.01, and ^*∗∗∗*^*p* < 0.001 compared with the control group.

**Figure 2:**
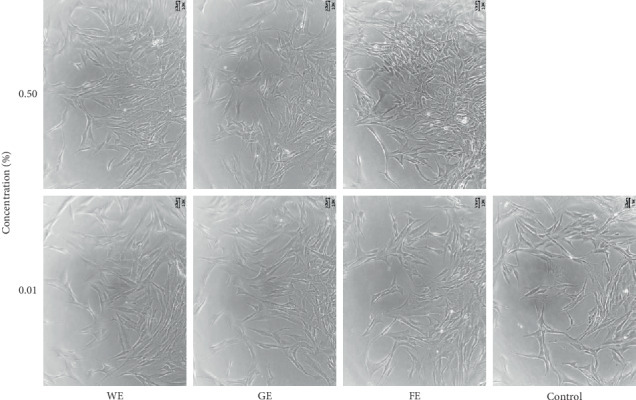
Comparison of the morphologies of human fibroblast cells on the third day after treatment with 0.01% and 0.5% of the three different extracts and comparison with the control (not treated). GE, gel juice extracted after peeling off the leaves of *A. vera*; WE, the water extract from the whole aerial parts of *A. arborescens* at 100°C for 24 hours; FE, fermentation of the whole aerial parts of *A. arborescens* grown with *L. plantarum* at 37°C for 4 days and further extracted by an ultrasonicator at 40 kHz for 1 hour at room temperature.

Figure 1 shows the cytotoxicity of the samples at various doses against human fibroblast cells. Interestingly, none of the three samples showed cytotoxicity over the entire range of concentrations tested, and the extracts even enhanced the mitochondrial activities of the cells in a concentration-dependent manner compared with the control (not treated with any of the extracts) because the dehydrogenase in active cells can easily breakdown MTT in the cellular mitochondria [36]. These results strongly imply that all the extracts from aloe do not exhibit cytotoxicity and improve cellular activities, which can result in the enhancement of the biological effects of the extracts based on the increased cell growth. Among the samples, the highest cellular activity (175% higher mitochondrial activity compared with the control) was achieved by the treatment with a dose of 0.5% of the extract of *A. arborescens* obtained after the lactic acid fermentation process (FE), followed by the extract of *A. arborescens* obtained after the water extraction process (WE, 140% activity) and the gel from *A. vera* (GE, 101% activity). However, a greater increase was not generally observed with doses above 0.5% even though an increase was observed with a sample dose of 1%. This result indicates that the dose limit for the aloe extracts was 0.5%, and this same upper limit was employed for the subsequent experiments. Interestingly, the enhancement observed with both the FE and WE was markedly higher than that obtained with the GE, and these results imply that the extracts from the aerial parts of the leaves of *A. arborescens* contained more bioactive substances than the GE extract, and the gel peeled off the inner parts of the leaves of *A. vera* did not have many active components. This finding was also supported by the result that the gels from the inner parts of *A. vera* leaves contain more than 95% water and only 4-5% high-molecular-weight polysaccharides, even though these polysaccharides and other minor components, such as anthraquinones, exhibit several biological activities [16, 41, 42]. Figure 1 also demonstrates the advantages of the fermentation process by showing that the FE substantially improved the mitochondrial activity of fibroblast cells to a greater extent than the WE and GE. This result was also confirmed in Figure 2, which compares the morphology of the fibroblast cells after 3 days of treatment with the extracts and that of the untreated control. The results clearly showed that treatment with both high and low doses of the FE resulted in denser cultures of fibroblast cells compared with the results obtained with the other two extracts and substantially denser cultures compared with the control, which was likely due to increased cell activation. In particular, an improved fibroblast morphology was observed after the high-dose treatment (0.5%) compared with the low-dose treatment (0.01%), and denser cultures were mostly observed after treatment with the FE compared with the other two extracts. This finding strongly indicates that the extracts from aloe positively influenced cell growth by boosting mitochondrial activity, and this effect, which was observed regardless of the aloe species, could also be enhanced by fermentation, which is a novel finding of this study. These findings indicate that the extract of *A. arborescens* exhibits better biological activity than gels from *A. vera*, and lactic acid fermentation increases this activity.

**Figure 3:**
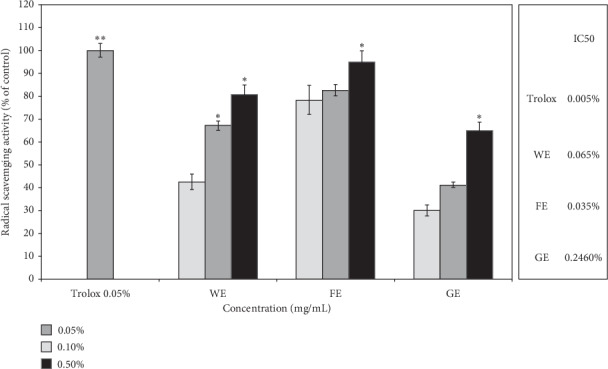
DPPH free radical scavenging activities of the three different extracts according to the treatment concentrations compared with 0.05% Trolox as a positive control. IC_50_ of each sample was also estimated in the table. GE, gel juice extracted after peeling off the leaves of *A. vera*; WE, the water extract from the whole aerial parts of *A. arborescens* at 100°C for 24 hours; FE, fermentation of the whole aerial parts of *A. arborescens* grown with *L. plantarum* at 37°C for 4 days and further extracted by an ultrasonicator at 40 kHz for 1 hour at room temperature. Error bars are mean values ± SD from triplicate separate experiments. The significance of difference was set to ^*∗*^*p* < 0.05, ^*∗∗*^*p* < 0.01, and ^*∗∗∗*^*p* < 0.001 compared with the control group.

To confirm the abovementioned assumption, the antioxidant activities of the three extracts were compared using a DPPH free radical scavenging assay. Approximately 95% antioxidant activity was observed after the treatment with the FE at the highest dose (0.5%), whereas 80% activity and 65% activity were detected after the treatment with the WE and GE at the same concentration, respectively. To be more easily compared, the results of estimating IC_50_ of each sample were also shown in Figure 3, which clearly proved that the FE had the highest antioxidant activities than the other two extracts. This result indicates that the antioxidant activity of the FE at a dose of 0.5% was very strong and close to that of 0.1% Trolox, which was used as the positive control, and the WE also showed relatively high antioxidant activity at the same concentrations. In contrast, GE did not show an impressive effect, even though it is considered to still exhibit fairly good antioxidant activity. Therefore, these results strongly indicate that the FE had higher antioxidant activity than the other two extracts, and this finding is supported by the greater improvement in cellular activity obtained with the FE, as shown in Figures 1 and 2. Based on the above-described results, it can be hypothesized that the *A. arborescens* extract obtained after the fermentation process might exhibit enhanced anti-skin wrinkling activities due to its high antioxidant activity because other studies have shown that high anti-skin wrinkling effects might be closely associated with high antioxidant activity [43, 44].

### 3.2. Anti-Skin Wrinkling Effects of the *A. arborescens* Extracts Obtained after Fermentation

**Figure 4:**
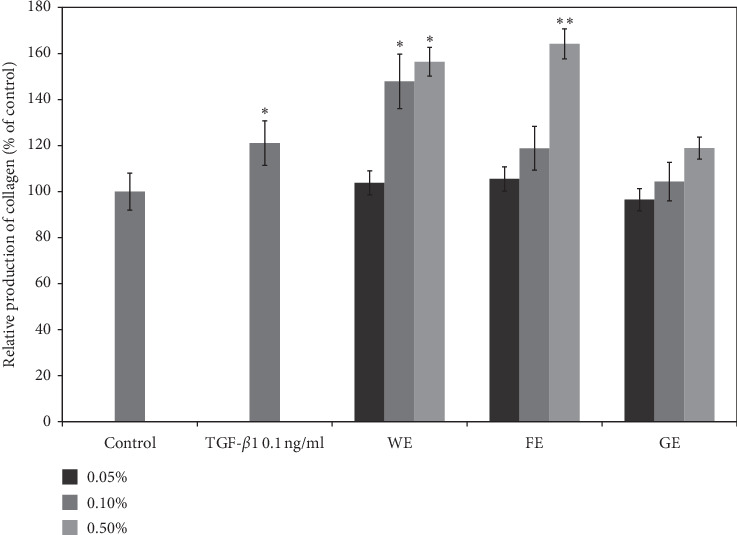
The production of collagen in human fibroblast cells after treatment with various concentrations of the three different extracts and 0.1 ng/ml TGF-*β*1 as a positive control. GE, gel juice extracted after peeling off the leaves of *A. vera*; WE, the water extract from the whole aerial parts of *A. arborescens* at 100°C for 24 hours; FE, fermentation of the whole aerial parts of *A. arborescens* grown with *L. plantarum* at 37°C for 4 days and further extracted by an ultrasonicator. Error bars are mean values ± SD from triplicate separate experiments. The significance of difference was set to ^*∗*^*p* < 0.05, ^*∗∗*^*p* < 0.01, and ^*∗∗∗*^*p* < 0.001 compared with the control group.

**Figure 5:**
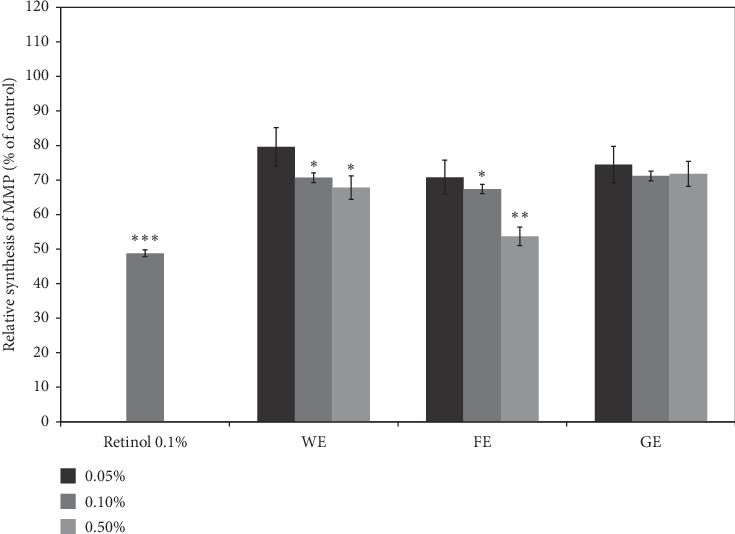
The inhibition of MMP-1 synthesis after treatment with various concentrations of three different extracts and 0.1% retinol as a positive control. GE, gel juice extracted after peeling off the leaves of *A. vera*; WE, the water extract from the whole aerial parts of *A. arborescens* at 100°C for 24 hours; FE, fermentation of the whole aerial parts of *A. arborescens* grown with *L. plantarum* at 37°C for 4 days and further extracted by an ultrasonicator at 40 kHz for 1 hour at room temperature. Error bars are mean values ± SD from triplicate separate experiments. The significance of difference was set to ^*∗*^*p* < 0.05, ^*∗∗*^*p* < 0.01, and ^*∗∗∗*^*p* < 0.001 compared with the control group.

Figures 4 and 5 illustrate the effects of the aloe extract obtained after lactic acid fermentation on the prevention of skin wrinkling compared with those of the other two extracts (the extract obtained after conventional water extraction and the gel extract of aloe). First, as shown in Figure 4, the effects of the extracts on collagen production by human fibroblast cells were observed and compared with those of TGF-*β*1, which was used as a positive control because TGF-*β*1 is closely related to skin regeneration and anti-wrinkling effects [45]. The results clearly confirm that the FE at the highest concentration (05%) is capable of greatly enhancing the synthesis of collagen by up to 170% compared with the control. At this same concentration, the WE and GE also showed 140% and 110% enhancement in collagen synthesis, respectively. The results also showed that the FE effectively increases collagen synthesis compared with that obtained after the treatment with 0.1 ng/mL TGF-*β*1 (an increase of only 120%). In general, the increase in collagen production obtained with all extracts was dependent on the concentration, which also confirmed that *A. arborescens* extracts can enhance collagen synthesis by human fibroblast cells. Interestingly, the FE more effectively increased the production of collagen at the highest dose tested compared with the lower dose tested and induced markedly improved effects compared with the other two extracts. Therefore, it is clear that the FE and WE exerted better anti-skin wrinkling effects than the GE from *A. vera*, even though most cosmetic applications of aloe, such as wound healing effects achieved by enhancing collagen synthesis by dermal cells, have involved the use of gels from *A. vera* [46–48]. We believe that this study provides the first demonstration that *A. arborescens* can increase collagen production, and this efficacy can be markedly improved by lactic acid fermentation.

The anti-skin wrinkling effects of *A. arborescens* were confirmed by investigating the inhibition of MMP-1 synthesis by the extracts, as shown in Figure 5; MMP-1 initiates the degradation of collagens produced by fibroblast cells, which can result in wrinkles on the surface of human skin [49]. As clearly shown in Figure 5, all of the aloe extracts can reduce the production of MMP-1; in addition, at a dose of 0.5%, the FE induced the highest inhibition of MMP-1 synthesis (48% of the control), whereas, at the same concentration, the WE and GE treatments resulted in only 28% and 20% inhibition, respectively. Moreover, the decrease in MMP-1 production observed with the FE was better than the 52% inhibition obtained with 0.1% retinol, which was used as the positive control because the recommended concentration of retinol for commercial creams is in the range of 0.075% to 1% [50]. Therefore, the inhibitory effects of the FE observed at a dose of 0.5% might be promising because retinol concentrations above 0.5% are considered effective in commercial products due to their lower stability under various light and temperature conditions, even though various side effects, such as the rapid inactivation of retinol and skin damage, have often been observed [50]. At the lowest concentration of 0.05%, the greatest inhibition of 25% was also obtained with the FE, followed by the WE (15% inhibition) and GE (12% inhibition). The inhibition of MMP-1 production was also found to be dependent on the concentration, similar to the trend found for collagen production (Figure 4). These results strongly support our hypothesis that the FE can better prevent the skin wrinkling process by both enhancing collagen production and inhibiting collagen degradation due to MMP-1 in fibroblast cells, whereas the simple extract of *A. arborescens* and the gel extract from *A. vera* can enhance the synthesis of collagens rather than inhibit the production of MMP-1. Therefore, these results strongly suggest that the FE can strongly exert enhanced anti-skin wrinkling effects compared with the other two aloe extracts (WE and GE), and the results obtained with the FE were similar to or even better than those obtained with *Carthamus tinctorius*, *Uncaria gambir*, and *Selaginella tamariscina*, which induce approximately 10–20% increases in collagen synthesis or inhibition of MMP-1 synthesis at dose ranges of 0.1 to 1% [51–53].

### 3.3. Concentrations of Barbaloin and Profiles of Polysaccharides with Different Molecular Weights in the Extracts

**Figure 6:**
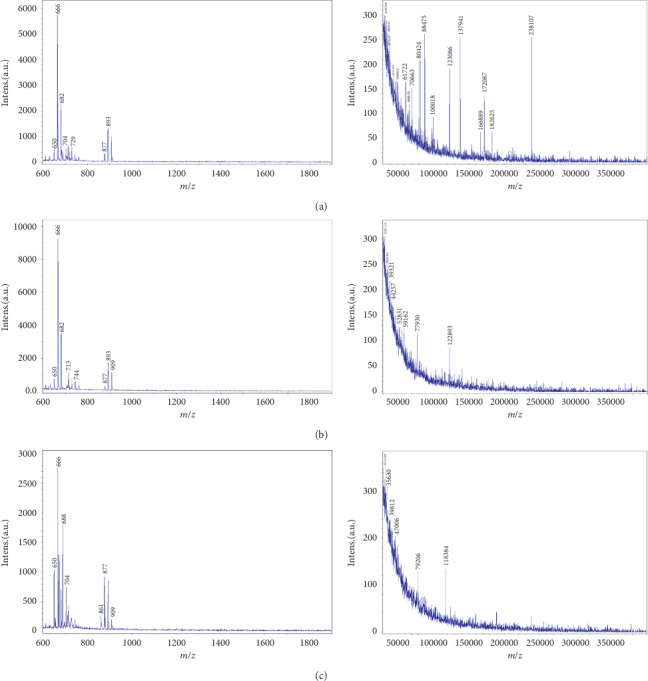
Comparison of the molecular weight distribution of the polysaccharides in the extracts from the three different processes obtained by MALDI-TOF analysis. (a) GE, gel juice extracted after peeling off the leaves of *A. vera*; (b) WE, the water extract from the whole aerial parts of *A. arborescens* at 100°C for 24 hours; (c) FE, fermentation of the whole aerial parts of *A. arborescens* grown with *L. plantarum* at 37°C for 4 days and further extracted by an ultrasonicator at 40 kHz for 1 hour at room temperature.

The above-described results clearly confirm that both the FE and the WE are capable of preventing human skin wrinkling. To elucidate the mechanisms underlying these results, the amounts of barbaloin, a bioactive component in aloe, were estimated, and the results are shown in Table 1. Barbaloin, as an aloin, is known to exist in most species of aloe and exhibits various biological activities, such as laxative, antioxidant, anticancer, anti-inflammatory, and antimicrobial activities [12, 54, 55]. The GE was found to have the lowest content of barbaloin (0.08 mg/g) because most aloins exist in the skin of aloe leaves [56], and the gel of *A. vera* investigated in this study (GE) was obtained by peeling off the skins. In contrast, both the WE and FE from *A. arborescens* contained fairly high amounts of barbaloin (10.21 mg/g and 9.38 mg/g, respectively) because these extracts were obtained by processing the full aerial parts of aloe. The amount of barbaloin in the WE was also similar to previously reported results, even though different values have been obtained depending on the culture conditions [56, 57]. In general, the whole aerial parts of *A. arborescens* rather than peeled-off skin are consumed, whereas *A. vera* has mostly been used as a gel; these different uses are based on the fact that *A. arborescens* is a relatively small and low plant grown on the ground compared with *A. vera*. The WE and FE used in this study were also obtained by processing the whole aerial parts of *A. arborescens*, which explains the higher amounts of barbaloin found in the WE and FE compared with the GE. This finding also supports the higher anti-skin wrinkling effects obtained from the WE or FE compared with the GE, which is likely due to their stronger antioxidant activities associated with their higher amounts of barbaloin. This study also showed that the content of barbaloin in the FE was slightly lower than that in the WE, which was likely obtained due to the slight breakage of barbaloin during the fermentation process. However, in most cases, the anti-skin wrinkling effects of the FE were markedly better than those of the WE. One possible explanation is the synergistic effects of barbaloin and the byproducts of lactic acid fermentation, and even though more detailed studies have been conducted, the published results show that the extracts obtained after fermentation exert better effects than those obtained after simple extraction processes [58, 59]. In addition to a possible synergistic effect associated with lactic acid fermentation, to more clearly explain the higher anti-skin wrinkling effects obtained with the FE, the distribution of polysaccharides in the extracts was also examined because many previous studies have indicated that most biological activities of aloe are likely caused by various types of polysaccharides [16, 60–62], and the results are shown in Figure 6 and Table 1. Moreover, as previously mentioned, the gels from aloe are mainly composed of water (95% of the total weight), and only 3-4% and less than 1% of the gels are composed of polysaccharides and 1% aloins, respectively [41, 63].

Therefore, the polysaccharides in aloe might also play an important role in preventing the skin wrinkling process, as proven in Figures 4 and 5. Interestingly, unlike the amounts of barbaloin shown in Figure 6, the profiles of polysaccharides of different MWs in the extracts were quite different. Specifically, very-high-MW polysaccharides were found in the gel that was squeezed directly from the inner parts of aloe, whereas the WE and FE obtained after water extraction and lactic acid fermentation, respectively, contained higher proportions of low-MW polysaccharides than the GE. Interestingly, the FE contained more lower-MW polysaccharides than the WE, which is likely due to the increased breakdown of high-MW polysaccharides that occurs during fermentation compared with conventional extraction processes. To more easily compare the MW profiles of the polysaccharides, the data in Figure 6 were quantified by matrix analysis, and the results are shown in Table 2. Table 2 clearly confirmed that 90% of the polysaccharides in the GE had a high MW in the range of 240,000 to approximately 90,000, and less than 10% were in the MW range of 600 to 700. Similar results have also been reported for gels from *A. vera* and other species with fairly high-MW polysaccharides in the range of 300,000 to 50,000 [8, 16, 64]. In contrast, approximately 90% of the polysaccharides in the WE had an MW less than 50,000, and the highest-MW polysaccharides were approximately 120,000. Interestingly, a major portion of the low-MW polysaccharides in the WE were in the range of 30,000. However, the molecular weight distributions of the polysaccharides in the FE were quite different from those in the other two extracts; specifically, approximately 20% of the polysaccharides were in the MW range of 600 to 900. These polysaccharide sizes were not found in the GE and WE even though the WE contained very small amounts of polysaccharides with a similar low MW. Notably, the portion of polysaccharides with an MW of 30,000–50,000 in the FE was 65%, whereas a value of 90% was found for the GE. These results clearly indicate that the fermentation process can further and/or more effectively breakdown high-MW polysaccharides to very small polysaccharides compared with conventional extraction processes that apply heat or other physical resources. These large amounts of small-MW polysaccharides in the FE would penetrate through the skin barrier more easily than larger-MW polysaccharides, which could result in more effective prevention of the skin wrinkling process through strong activation of the cellular metabolism of human fibroblasts associated with high amounts of barbaloin. Therefore, these results can explain why the FE consistently exerted the strongest anti-skin wrinkling effects and higher antioxidant activities compared with the WE, and most of the activities of the FE were markedly higher than those of the GE. Previously reported data also support our results that relatively low-MW polysaccharides in aloe can induce increased anti-inflammatory effect due to high antioxidant activities [65]. We believe that this work provides the first clear demonstration that the low-MW polysaccharides in *A. arborescens* extracts obtained after lactic acid fermentation can improve the anti-skin wrinkling effects. The results also confirm that the FE contains sufficient barbaloin to exert anti-skin wrinkling effects, and the barbaloin content in the FE was similar to that found in the WE but different from that found in the GE. Therefore, it can be concluded that the FE exerts strong anti-skin wrinkling effects associated with high antioxidant activity, which is likely due to synergistic effects between barbaloin and low-MW polysaccharides associated with lactic acid bacteria, and these effects of the FE were markedly stronger than those of the WE and GE.

## 4. Conclusion

This study provides the first demonstration that the extract obtained after the lactic acid fermentation of *Aloe arborescens* (FE) exerts anti-skin wrinkling effects by effectively enhancing the production of collagen and inhibiting the synthesis of MMP-1 in human fibroblast cells. The effects of the FE were also found to be closely associated with its high antioxidant activity. Interestingly, the FE greatly activated the growth of human fibroblast cells compared with the other two extracts (WE and GE), which indicated that the enhancement of fibroblast cell growth induced by the FE could also be correlated with its anti-skin wrinkling effects. The results also provide the first demonstration that the extract obtained with the conventional water extraction process of *A. arborescens* (WE) exhibited fairly good anti-skin wrinkling efficacy, although this efficacy was lower than that of the FE. The results of this study also confirmed that the anti-skin wrinkling activities of *A. arborescens* are markedly stronger greater than those of the gel peeled off from *A. vera* leaves (GE), which is the most widely utilized aloe-related material used for cosmetic applications to date. These strong anti-skin wrinkling effects of the FE were confirmed through the finding that these effects were similar to those observed after the treatment with retinol at a concentration of 0.1%, which is the minimum recommended concentration of retinal for use in commercial products with anti-skin wrinkling efficacy. The results clearly showed that the MW distribution of the polysaccharides in the FE differed from that of the WE and was marked more different than that of the GE. Specifically, 20% and 65% of the FE consisted of very small polysaccharides (in the MW range of 600 to 900) and polysaccharides in the MW range of 30,000 to 50,000, whereas 90% of the WE consisted of polysaccharides in the MW range of 30,000 to 50,000. These low-MW polysaccharides were not found in the GE or the aloe extracts investigated in previous studies because fermentation by lactic acid bacteria can effectively breakdown high-MW polysaccharides to achieve enhanced anti-skin wrinkling effects. In general, the majority of the polysaccharides in aloe gels are in the MW range of 200,000 300,000, and polysaccharides with similar MWs were also observed in the GE investigated in this study. The results also showed that the content of barbaloin, which is an aloin that is an active component, in the FE was similar to that in the WE, whereas only trace amounts were found in the GE. Based on these results, the *A. arborescens* extract obtained after lactic acid fermentation can exert enhanced anti-skin wrinkling effects, possibly due to the synergistic effects of low-MW polysaccharides and barbaloin. These effects might also be due to the easy uptake of low-MW polysaccharides by fibroblast cells, which would result in high activation of cellular metabolism in human fibroblasts. Conclusively, this work clearly demonstrates the effects of lactic acid fermentation on enhancing the anti-skin wrinkling effects of *A. arborescens*, which, as previous studies have shown, exerts anti-skin wrinkling effects. However, further investigation of the downregulation of genes and proteins related to skin wrinkling should be performed to confirm the detailed mechanisms underlying the anti-skin wrinkling activities of the FE associated with lactic acid fermentation.

## Figures and Tables

**Table 1:** The concentrations of barbaloin in the aloe extracts from the three different processes.

Extracts	Contents (mg/g)
Gel extract (GE)^*∗*^	0.08 ± 0.02
Water extract (WE)^*∗∗*^	10.21 ± 0.72
Fermentation extract (FE)^*∗∗∗*^	9.38 ± 0.13

^*∗*^GE: the gel juice extracted after peeling off the leaves of *Aloe vera*. ^*∗∗*^WE: the extract obtained by hot water extraction of *Aloe arborescens* at 100°C for 24 hours. ^*∗∗∗*^FE: the lactic acid fermentation of *Aloe arborescens* at 37°C for 4 days further extracted by an ultrasonicator at 40 kHz for 1 hour at room temperature.

**Table 2:** Comparison of the molecular weight (MW) profiles of the polysaccharides in the aloe extracts from the three different processes.

GE^*∗*^		WE^*∗∗*^		FE^*∗∗∗*^	
MW	% composition	MW	% composition	MW	% composition
238107.229	21.25	118384.358	7.17	122893.982	4.43
172087.419	12.46	79206.108	4.73	77930.07	8.18
138034.792	11.27	47006.643	6.04	52831.109	6.42
137941.051	19.80	39604.995	6.82	44257.96	5.78
123086.755	13.87	38734.689	5.79	44161.622	7.07
88475.398	14.65	34507.023	6.26	42536.868	5.48
729.656	1.15	34310.378	4.58	35815.964	5.59
713.562	1.26	34186.908	6.36	35384.405	5.67
682.314	1.11	33145.206	9.79	31084.446	5.59
666.341	3.19	32952.698	4.76	30577.379	6.18
—	—	32014.32	7.66	30518.263	6.40
—	—	30526.307	6.90	30132.334	7.04
—	—	30262.916	5.24	30092.126	5.61
—	—	30216.016	5.03	666.429	9.28
—	—	30135.666	5.39	893.346	0.86
—	—	30110.233	6.28	877.365	0.89
—	—	682.294	0.39	713.598	1.51
—	—	666.324	0.82	710.640	1.40
—	—	—	—	688.328	1.70
—	—	—	—	672.360	1.19
—	—	—	—	666.343	2.78
—	—	—	—	650.370	0.94

^*∗*^GE: the gel juice extracted after peeling off the leaves of *Aloe vera*. ^*∗∗*^WE: the extract obtained by hot water extraction of *Aloe arborescens* at 100°C for 24 hours. ^*∗∗∗*^FE: the lactic acid fermentation of *Aloe arborescens* at 37°C for 4 days further extracted by an ultrasonicator at 40 kHz for 1 hour at room temperature.

## Data Availability

The data used to support the findings of this study will be provided by author's consent upon request.

## References

[1] Haller J. S. (1990). A drug for all season medical and pharmacological history of aloe. *Bulletin of the New York Academy of Medicine*.

[2] Das A., Mukherjee P., Jha T. B. (2010). High frequency micropropagation of *Aloe vera* L. Burn. F. as a low cost option towards commercialization. *Plant Tissue Culture and Biotechnology*.

[3] Cui Y., Ye Q., Wang H., Li Y., Yao W., Qian H. (2014). Hepatoprotective potential of *Aloe vera* polysaccharides against chronic alcohol-induced hepatotoxicity in mice. *Journal of the Science of Food and Agriculture*.

[4] Dagne E., Bisrat D., Viljeon A., Wyk B.-E. V. (2000). Chemistry of aloe species. *Current Organic Chemistry*.

[5] Crewe J. E. (1985). Aloe in the treatment of burn and scalds. *Minnesota Medicine*.

[6] Femenia A., Pascual P. G., Simal S., Rosselló C. (2003). Effects of heat treatment and dehydration on bioactive polysaccharides acemannan and cell wall polymers from *Aloe barbadensis* Miller. *Carbohydrate Polymers*.

[7] Lee L. M., Haggers J. P., Robson M. C. (1980). The therapeutic efficacy of *Aloe vera* cream in thermal injuries: two case report. *Journal of the American Animal Hospital Association*.

[8] Rodríguez E. R., Martín J. D., Romero C. D. (2010). *Aloe vera* as a functional ingredient in foods. *Critical Reviews in Food Science and Nutrition*.

[9] Ferreira M., Teixeira M., Silva E., Selores M. (2007). Allergic contact dermatitis to *Aloe vera*. *Contact Dermatitis*.

[10] Davis R. H., Kabbani J. M., Mara N. P. (1987). *Aloe vera* and wound healing. *Journal of the American Podiatric Medical Association*.

[11] Curto E. M., Labelle A., Chandler H. L. (2014). *Aloe vera*: an *in vitro* study of effects on corneal wound closure and collagenase activity. *Veterinary Ophthalmology*.

[12] Alves D. S., Péres-Fons L., Estepa A., Micol V. (2004). Membrane-related effects underlying the biological activities of the anthraquinones emodlin and barbaloin. *Biochemical Pharmacology*.

[13] Yen G.-C., Duh P.-D., Chuang D.-Y. (2000). Antioxidant activities of anthraquinones and anthrone. *Food Chemistry*.

[14] Park M.-Y., Kwon H.-J., Sung M.-K. (2009). Evaluation of aloin and aloe-emodin as anti-inflammatory agents in Aloe by using murine macrophages. *Bioscience, Biotechnology, and Biochemistry*.

[15] Eshun K., He Q. (2004). *Aloe vera*: a valuable ingredients for the food, pharmaceutical and cosmetic industries-a review. *Critical Reviews in Food Science and Nutrition*.

[16] Pugh N., Rodd S. A., Elsohly M. A., Pasco D. S. (2001). Characterization of aloeride, a new high molecular weight polysaccharides from *Aloe vera* with potent immunostimulatory activity. *Journal of Agricultural and Food Chemistry*.

[17] Chang X. L., Wang C., Feng Y., Liu Z. (2006). Effects of heat treatments on the stabilities of polysaccharides substances and barbaloin in gel juice from *Aloe vera* Miller. *Journal of Food Engineering*.

[18] Gutterman Y., Chauser-Volfson E. (2006). Changes in secondary phenolic metabolites during storage as an aqueous suspension in comparing with the contents in harvested *Aloe arborescens* leaves. *International Journal of Food Science and Technology*.

[19] Andersen F. A. (2007). Final report on the safety assessment of *Aloe andogensis* extract, *Aloe arborescens* leaf extracts, *Aloe arborescens* leaf juice, *Aloe arborescens* leaf protoplasts, *Aloe barbadensis* leaf juice, *Aloe barbdensis* leaf extract and *Aloe ferox* leaf juice extracts. *International Journal of Toxicology*.

[20] Yagi A., Harada N., Shimomura K., Nishioka I. (1987). Bradykinn-degrading glycoprotein in *Aloe arborescens* var. natalensis. *Planta Medica*.

[21] Nazeam J. A., Gad H. A., Esmat A., El-Hefnawy H. M., Singab A. N.-B. (2017). *Aloe arborescens* polysaccharides: *in vitro* immunomodulation and potential cytotoxic activity. *Journal of Medicinal Food*.

[22] Kong B.-M., Park M.-J., Min J.-W. (2008). Physico-chemicial characteristics of white, fermented and red ginseng extracts. *Journal of Ginseng Research*.

[23] Perdigón G., Fuller R., Raya R. (2001). Lactic acid bacteria and their effect on the immune system. *Current Issues in Intestinal Microbiology*.

[24] Yang M. C., Sang W. J., Jin Y. M. (2011). Analysis of constituents in Sipjundaebo-tangs fermented by lactic acid bacteria. *Korean Journal of Microbiology and Biotechnology*.

[25] Kang H. J., Park K. M. (2001). GRAS notification process in the US. *The Microorganisms and Industry*.

[26] Kang Y.-M., Hong C.-H., Kang S.-H. (2020). Anti-photoaging effect of plant extract fermented with *Lactobacillus buchneri* on CCD-986sk fibroblast and HaCaT keratinocytes. *Journal of Functional Biomaterials*.

[27] Sharma D., Kober M.-M., Bowe W. P. (2016). Anti-aging effects of probiotics. *Journal of Drugs in Dermatology*.

[28] Lee D. E., Huh C.-S., Ra J. (2015). Clinical evidence of effects of *Lactobacillus plantarum* HY7714 on skin aging: a randomized, double blind, placebo-controlled study. *Journal of Microbiology and Biotechnology*.

[29] Farage M. A., Miller K. W., Elsner P., Maibach H. I. (2007). Structural characteristics of the aging skin: a review. *Cutaneous and Ocular Toxicology*.

[30] Kim Y. S., Lee D. E., Park S. D. (2014). Oral administration of *Lactobacillus plantarum* HY7714 protects hairless mouse against ultraviolet B-induced photoaging. *Journal of Microbiology and Biotechnology*.

[31] Lee X.-M., Lee H. A., Kwon M., Park E.-S., Park K.-Y. (2016). Probiotic effects of *Lactobacillus plantarum* strains isolated from kimchi. *Journal of the Korean Society of Food Science and Nutrition*.

[32] Tian J., Liu J., Zhang J., Hu Z., Chen X. (2003). Fluorescence studies on the interactions of barbaloin with bovine serum albumin. *Chemical & Pharmaceutical Bulletin*.

[33] The Food Code for Functional Foods, The Standard Protocols of Measuring Anthraquinone as Anhydrous Barbaloins in Foods (Method I), Section 8.1.3, The Korea Food and Drug Administration (KFDA), 2020

[34] Karas M., Bahr U. (1990). Laser desorption ionization mass spectrometry of large biomolecules. *TrAC Trends in Analytical Chemistry*.

[35] Paz B., Riobó P., Franco J. M. (2011). Preliminary study for rapid determination of phycotoxins in microalgae whole cells using matrix assisted laser desorption/ionization time-of-flight mass spectrometry. *Rapid Communications in Mass Spectrometry*.

[36] Mosmann T. (1983). Rapid colorimetric assay for cellular growth and survival: application to proliferation and cytotoxicity assays. *Journal of Immunological Methods*.

[37] Kim J.-S., Seo Y.-C., Choi W.-Y. (2011). Enhancement of antioxidant activities and whitening effect of *Acer mono* sap through nano encapsulation processes. *Korean Journal of Medicinal Crop Science*.

[38] Dietz B. M., Kang Y.-H., Liu G. (2005). Xanthohumol isolated from *Humulus lupulus* inhibits menadione-induced DNA damage through induction of quinone reductase. *Chemical Research in Toxicology*.

[39] Kim T. Y., Leem K.-H. (2015). Effects of *Draconis resina* on the collagenase activities and the procollagen synthesis in Hs68 human fibroblasts, and tyrosinase activity. *The Korea Journal of Herbology*.

[40] Yoshikawa H., Oyamada I., Usuku G. (1987). An assay of collagenase activities using enzyme-linked immunosorbent assay for mammalian collagens. *Analytical Biochemistry*.

[41] Reynolds T., Dweck A. C. (1999). *Aloe vera* leaf gel: a review update. *Journal of Ethnopharmacology*.

[42] Agarwala O. P. (1997). Whole leaf aloe gel vs. standard aloe gel. *Drug and Cosmetics Industry*.

[43] Kim N.-M., Koo B.-S., Lee S.-K., Hwang E.-I., So S.-H., Do J.-H. (2007). Effect of Korean red ginseng on collagen biosynthesis and MMP-I activity in human dermal fibroblast. *Journal of Ginseng Research*.

[44] Pentland A. P., Shapiro S. D., Welgus H. G. (1995). Agonist-induced expression of tissue inhibitor of metalloproteinases and metalloproteinases by human macrophages is regulated by endogenous prostaglandin E_2_ synthesis. *Journal of Investigative Dermatology*.

[45] Woo C. Y., Kim D. C. (2017). Skin regeneration, anti-wrinkle, whitening and moisturizing effects of cheongsangbangpung-tang aqueous extracts with cytotoxicity. *The Journal of Korean Obstetrics and Gynecology*.

[46] Heggars J. P., Kucucelelsi A., Listengarten D. (1996). Beneficial effect of Aloe on wound-healing in an excisional wound model. *The Journal of Alternative and Complementary Medicine*.

[47] Takahashi M., Kitamoto D., Asikin Y., Takara K., Wada K. (2009). Liposomes encapsulating *Aloe vera* leaf gel extract significantly enhance proliferation and collagen synthesis in human skin cell lines. *Journal of Oleo Science*.

[48] Chitha P., Sajithlal G. B., Chanderakasan G. (1998). Influence of *Aloe vera* on collagen characteristics in healing dermal wounds in rats. *Molecular and Cellular Biochemistry*.

[49] Park S.-H., Lee K.-H., Han C.-S., Kim K.-H., Kim Y. H. (2010). Inhibitory effects of *Carex humilis* extract on elastase activityand matrix metalloproteinase-1 expression. *Journal of Society of Cosmetic Scientists of Korea*.

[50] Kim B.-H. (2010). Safety evaluation of skin wrinkling effects of retinoids on skin. *Toxicological Research*.

[51] Kim M. J., Kim J. Y., Choi S. W. (2004). Anti-wrinkling effect of sufflower seed extract (I). *Journal of Society of Cosmetic Scientists of Korea*.

[52] Lee J. M., Kim D.-H., Kim E.-W. (2017). Matrix metalloproteinase-1 suppression and type-1 procollagen synthesis promoting effects of *Uncaria gambir*. *Korean Journal of Food Preservation*.

[53] Sim G. S., Kim J. H., Park S. M. (2004). Effect of the *Selaginella tamariscina* extract on antioxidant and inhibition of matrix metalloproteinase-1 in human skin fibrlblasts. *Yakhak Hoeji*.

[54] Huichun Z., Ruiqin F., Xiaoguang D., Linpei J. (1998). Study of the Eu (III)-barbaloin-Ctab system by fluorescence and determination of barbaloin. *Analytical Letters*.

[55] Groom Q., Reynolds T. (1987). Barbaloin in Aloe species. *Planta Medica*.

[56] Esua M. F., Rauwald J.-W. (2006). Novel bioactive maloyl glucans from *Aloe vera* gel: isolation, structure elucidation and *in vitro* bioassays. *Carbohydrate Research*.

[57] Chauser-Volfson E., Gutterman Y. (1996). The barbaloin content and distribution in *Aloe arborescens* leaves according to the leaf part, age, position, and season. *Israel Journal of Plant Sciences*.

[58] Tsai C.-C., Chan C.-F., Huang W.-Y. (2013). Applications of *Lactobacillus rhamnosus* spent culture supernatant in cosmetic antioxidation, whitening and moisture retention applications. *Molecules*.

[59] Ryu I. H., Kwon T. O. (2012). Enhancement of piperidine alkaloid contents by lactic acid fermentation of Mulberry leaves (*Morus alba* L). *Korean Journal of Medicinal Crop Science*.

[60] Im S.-A., Oh S.-T., Song S. G. (2005). Identification of optimal molecular size of modified *Aloe* polysaccharides with maximum immunomodulatory activity. *International Immunopharmacology*.

[61] Femenia A., Sánchez E. S., Simal S., Rosselló C. (1999). Compositional features of polysaccharides from *Aloe vera* plant tissues. *Carbohydrate Polymers*.

[62] Eberendu A. R., Luta G., Edwards J. A. (2005). Quantitative colorimetric analysis of aloe polysaccharides as a measure of *Aloe vera* quality in commercial products. *Journal of AOAC International*.

[63] Grindlay D., Reynolds T. (1986). The *Aloe vera* phenomenon: a review of the properties and modern uses of the leaf parenchyma gel. *Journal of Ethnopharmacology*.

[64] Tabandeh M. R., Oryan A., Mohammadalipour A. (2014). Polysaccharides of *Aloe vera* induce the skin wound repair of rat. *International Journal of Biological Macromolecules*.

[65] Davis R. H., Leitner M. G., Russo J. M., Byrne M. E. (1989). Anti-inflammatory activity of *Aloe vera* against a spectrum of irritants. *Journal of the American Podiatric Medical Association*.

